# Magnetic Hydrogel for Cartilage Tissue Regeneration as well as a Review on Advantages and Disadvantages of Different Cartilage Repair Strategies

**DOI:** 10.1155/2022/7230354

**Published:** 2022-04-08

**Authors:** Parto Babaniamansour, Maryam Salimi, Farid Dorkoosh, Maryam Mohammadi

**Affiliations:** ^1^Department of Biomedical Engineering, AmirKabir University of Technology, Tehran, Iran; ^2^Student Research Committee, Shiraz University of Medical Sciences, Shiraz, Iran; ^3^Bone and Joint Diseases Research Center, Department of Orthopedic Surgery, Shiraz University of Medical Sciences, Shiraz, Iran; ^4^Medical Biomaterial Research Center (MBRC), Tehran University of Medical Sciences, Tehran, Iran; ^5^Department of Pharmaceutics, Tehran University of Medical Sciences, Tehran, Iran; ^6^Department of Biomedical Engineering, University of Isfahan, Isfahan, Iran

## Abstract

There is a clear clinical need for efficient cartilage healing strategies for treating cartilage defects which burdens millions of patients physically and financially. Different strategies including microfracture technique, osteochondral transfer, and scaffold-based treatments have been suggested for curing cartilage injuries. Although some improvements have been achieved in several facets, current treatments are still less than satisfactory. Recently, different hydrogel-based biomaterials have been suggested as a therapeutic candidate for cartilage tissue regeneration due to their biocompatibility, high water content, and tunability. Specifically, magnetic hydrogels are becoming more attractive due to their smart response to magnetic fields remotely. We seek to outline the context-specific regenerative potential of magnetic hydrogels for cartilage tissue repair. In this review, first, we explained conventional techniques for cartilage repair and then compared them with new scaffold-based approaches. We illustrated various hydrogels used for cartilage regeneration by highlighting the magnetic hydrogels. Also, we gathered in vitro and in vivo studies of how magnetic hydrogels promote chondrogenesis as well as studied the biological mechanism which is responsible for cartilage repair due to the application of magnetic hydrogel.

## 1. Introduction

Cartilage injuries occur due to degenerative disease and surgical and traumatic injuries [1, 2]. The hyaline cartilage legions account for the highest rate of world disability [3]; only in the USA, more than 250000 patients require knee arthroplasty for articular cartilage each year [4]. International Cartilage Repair Society assessed that more than 60% of the patients undergoing knee arthroscopy demonstrated cartilage damage [4, 5]. Furthermore, more than 50% of the world population older than 65 years old suffer from osteoarthritis (OA) which is traditionally characterized as cartilage damage [6, 7]. Articular cartilage disorder occurs in conjunction between the bones and worsens over time by constant mechanical degeneration and loss of cartilage tissue, resulting in osteoarthritis [3]. Cartilage has a limited ability for self-repair due to its low vascularity which constraints the replicative ability of the chondrocyte [8, 9]. In contrast with the bone tissue, the cartilage has a few cells and metabolic activity, and the limited number of these cells, if any, is specialized in cartilage remodeling [9]. Without cells and mediators or the lack of accessibility to abundant nutrients even small cartilage defect does not have the innate ability to achieve sufficient healing [10]. Furthermore, the lack of specific diagnostic biomarkers is another reason that makes regeneration of the cartilage a clinical issue for decades [11].

Traditional techniques for curing articular cartilage defects include microfracture, osteochondral autographs, and allographs [12]. Shortcoming reported for current treatment includes the requirement of secondary surgery for osteochondral autographs or immune rejection and transmission of donor pathogen after allographs [13, 14]. Lack of efficient regeneration technique for articular cartilage necessitates tissue engineering approaches combining scaffolds, cells, and growth factors [13]. Cartilage formation is a complex cascade that is influenced by a cocktail of cytokines and growth factors that guide inflammatory cells to the injury site [15]. Hydrogels inducing chondrogenesis via chemical agents can be good therapeutic agents; however, they are not still ideal for clinical application. The limitations associated with the use of growth factors and chemical agents for cartilage tissue regeneration include protein denaturation, side effects, and off-target effects. New studies revealed that the fabrication of novel magnetic hydrogels that ensure cell function and sufficient biomechanical properties to the regenerative environment can be useful for cartilage tissue regeneration [16]. The electromagnetic field (EMF) was previously shown to promote osteogenic differentiation of mesenchymal stem cells (MSC) [17]. It is hypothesized that EMF can also trigger chondrogenic differentiation of stem cells which can be used for the preparation of novel cartilage tissue scaffolds [18–21].

The application of the magnetic hydrogel for cartilage tissue regeneration is still at the beginning of the road and much has not been done in this area. There is a need for a detailed study of magnetically induced chondrogenesis and magnetic hydrogels. In this review, a study was done on the fabrication of magnetic hydrogels that induce chondrogenesis. Furthermore, the influence of magnetically labeled stem cells in enhancing the cartilage matrix synthesis was investigated. In addition, the results of in vitro and in vivo studies of magnetic hydrogel for articular cartilage defects were discussed.

A variety of natural and synthetic polymers have been studied for the fabrication of hydrogels for cartilage regeneration. Commonly used polymers for cartilage tissue regeneration are summarized in Table 1. Collagen, hyaluronic acid (HA), and chondroitin sulfate (CS) are widely used for cartilage regeneration since they can mimic natural cartilage ECM and induce chondrogenesis [22, 23]. Lack of mechanical properties and quick degradation are the shortcomings of most hydrogel scaffolds [24, 25]. Insufficient mechanical or chemical properties can be rectified by crosslinking the polymer with other materials in order to obtain a hybrid polymer [20]. However, a hybrid hydrogel-based scaffold should be designed and manufactured in a way to overcome different shortcomings other than mechanical properties. For instance, the low number of chondrocytes in the cartilage is a limitation that should be addressed by a scaffold that can enhance chondrogenic cell differentiation [26]. Lack of chondrogenesis in the conventional hydrogel-based scaffolds necessitates the fabrication of smart hydrogels that can respond to certain cues. The desire to remotely regulate the cartilage physical and chemical microenvironment has attracted attention to the fabrication of magnetically responsive hydrogels [27]. Therefore, fabricating magnetic hydrogels that can create a microenvironment that ensures chondrocyte cell viability and proliferation and enhances chondrogenesis can be a good cartilage tissue engineering approach.

## 2. Cartilage Structure

**Figure 1:**
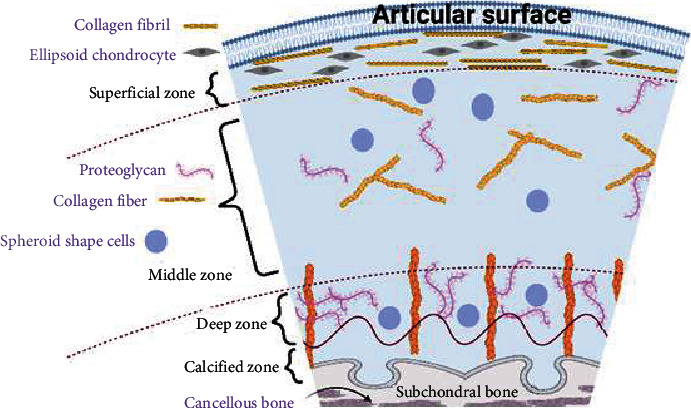
Schematic of the articular cartilage.

Hyaline cartilage is a nonvascularized connective tissue and the most abundant cartilage type of the body which is found in the costal cartilage, trachea, and articular cartilage [16, 37]. The articular cartilage has a highly organized but heterogeneous structure that is composed of small numbers of chondrocytes and a multicomponent matrix [37, 38]. Between 70 and 85% of the articular cartilage is water and 30% of the dry tissue is proteoglycan [38]. As demonstrated in Figure 1, the articular structure of the cartilage is categorized into four zones the superficial, middle, deep, and calcified zone [39]. The superficial zone is the thinnest layer which is composed of ellipsoid chondrocyte and collagen fibrils [40]. Collagen fibers are packed in a superficial layer and are oriented parallel to the articular surface [41]. The superficial zone has the highest water content and provides a gliding surface to the cartilage as well as protection from the synovial fluid immune cells [40, 41]. The middle or transitional zone has a relative proportion between 40 and 60%, while the superficial layer is 10-20% of the articular cartilage [42, 43]. This zone is composed of spheroid-shaped cells and thick collagen fibers that are oriented obliquely [40]. The middle zone induces compatibility between shear forces of the superficial layer and compressive forces of the deeper zone [40]. Deep or radial zone is composed of spheroidal-shaped chondrocytes as well as the highest concentration of proteoglycans [44]. Chondrocytes are larger and collagens are thicker in the deep zone as compared with the other zones [45]. Collagens are oriented perpendicularly to facilitate load distribution and resist compression [40]. The glycosaminoglycan (GAG) amount is usually lower in the superficial zone, enhances in the middle layer, and drops in the deep layer towards the tidemark [42]. Extension of collagen from deep zone to calcified zone preserves cartilage and bone integrity [40]. The calcified zone is the boundary between cartilage and subchondral bone and provides a barrier to diffusion from blood vessels supplying the bone [40].

## 3. Cartilage Injuries and Repair

Several pathological conditions such as infectious disease, osteoarthritis, cancer, or traumatic injuries can lead to cartilage defects. Articular cartilage has a low intrinsic capacity for repair so treatment of defects of articular cartilage whether from trauma or degenerative disease is a significant challenge for orthopedic surgeons [46, 47]. Although many techniques have been introduced for the treatment of cartilage defects, not enough is known about the suitable treatment modality for a particular lesion [48]. Due to the fact that no universally accepted system is established to define a lesion, it may be hard to decide which lesion requires treatment and discuss the results of treatment of focal cartilage impairments [48]. Two major issues when repairing the articular cartilage are filling the defect void with the material of similar mechanical properties with cartilage and inducing integration between repair tissue and native cartilage [49]. Current most common strategies for healing cartilage defects include microfracture, osteochondral transfer, autologous chondrocyte implantation (ACI), and scaffolds.

### 3.1. Microfracture Technique for Cartilage Repair

Microfracture is a safe, effective, minimally invasive, and marrow-stimulating technique that aims at triggering vascular response to injury [50]. This technique includes abrading the tidemark and creating small holes perpendicular to the subchondral bone plate to allow bleeding into the defect. In this technique, the stem cells extravasate from the subchondral area to the chondral defect and they differentiate to the fibrocartilage tissue [50]. At the same time, the body's own tissue healing process maintains the integrity of marrow clot and subchondral plate, inducing durable cartilage repair [51]. Microfractures usually result in a fibrous-fibro hyaline unstructured repair tissue that lacks the biomechanical and viscoelastic features of hyaline cartilage [52–54]. Of note, collagen scaffolds are commonly inserted postmicrofracture to augment marrow stimulation [55]. Different factors are influential in determining the success of the microfracture technique such as the dimension and location of the defect as well as the age and gender of the patient [56].

### 3.2. Osteochondral Transfer

When the articular defect size is bigger than 15 mm, the transplantation of autogenic and allogenic tissue is preferred over marrow-stimulating techniques [57]. This technique introduces a new hyaline cartilage surface, while microfracture yields a fibrocartilage repair [58]. The source of tissue for the osteochondral transfer may be either autogenic or allogenic. Allogenic tissue is obtained from cadaveric tissues, but the autogenic tissue can provide better mechanical stability and biocompatibility. Although articular cartilage is located in an immunologically privileged position, the immune response is still a major concern with the osteochondral transfer technique [49]. Another disadvantage of this technique is donor-site morbidity after osteochondral transfer due to the cell death at the wound margin which results in tissue degeneration over time [27].

### 3.3. Autologous Chondrocyte Implantation (ACI)

ACI is the first cell-based biological approach to the treatment of grade III and grade IV cartilage lesions [52, 59] that introduces end-differentiated chondrocytes into the prepared defect, resulting in the formation of a hyaline-like repair tissue [60, 61]. ACI was initially used for the treatment of focal chondral injury and later this technique utilized various 3D polymeric scaffolds [62]. The ACI technique includes creating small biopsy of the hyaline cartilage, extracting chondrocytes from a less-weight-bearing area of articular surface, and culturing the cells in vitro [63]. Then, chondrocyte cells are expanded in vitro to enhance the number of cells to provide enough number to fill a focal articular defect [64]. Once the chondrocyte cell population achieved a certain level in vitro, they are implanted into the cartilage defect. After chondrocytes are implanted into the articular defect, they start to produce cartilage matrix that gradually fills out the cartilage defect. This is similar to the mesenchymal condensation that happens during the limb formation [63, 65]. The periosteal graft may cause complications after ACI which can be addressed to some extent by using collagen scaffolds instead of periosteal patches [55].

### 3.4. Scaffold

Three-dimensional scaffolds are becoming popular because they are cost-effective, time-efficient, and require a single-stage procedure. Furthermore, they provide a high capacity for cell attachment as well as adjustability for having appropriate mechanical properties [66, 67]. Furthermore, scaffolds have been promising in improving the conventional cartilage repair techniques such as microfracture and ACI by promoting chondrocyte transfer and graft incorporation to enhance hyaline cartilage [55].

Since the beforementioned treatments (microfracture, osteochondral transfer, and ACI) have limitations, the hopes are in scaffold tissue engineering. However, because the chemical and mechanical properties of the cartilage tissue are not consistent through the entire tissue, it is complicated to create a scaffold that can fully mimic the natural cartilage [68]. Therefore, multiphasic scaffolds are more preferred than monophasic scaffolds since they have several layers with controlled properties to imitate the local microenvironment of cartilage tissue [69]. For example, Nguyen et al. [70] fabricated three-layer polyethylene glycol-based scaffold with the chemical gradient of chondroitin sulfate, matrix metalloproteinase-sensitive peptides, and hyaluronic acid by being inspired by the depth-dependent morphology of the cartilage. They showed that MSCs within the scaffold have undergone chondrogenic differentiation and also matrix production profile was compatible with the specific zone of the articular cartilage [70]. Similarly, Liu et al. [71] developed BMDS-laden 3D-bioprinted multilayer scaffold and studied its effect on the animal model of osteochondral defect repair. They observed enhanced collagen type II production as well as decreased inflammatory cytokines in the injury site which has resulted in increased chondrogenesis [71].

Different materials have been suggested for cartilage regeneration purposes (synthetic or natural) and they can be used in various physical forms of fibers, meshes, and hydrogels. Hyaluronan and collagen-based scaffolds are polymers widely used for the fabrication of cartilage scaffolds due to their similarity to the natural cartilage tissue [49, 64]. Scaffolds are designed to be chondro-conductive and they can be used with or without cells. Although chondrocytes are the most commonly used cells for tissue engineering purposes, the potential of MSCs for cartilage regeneration is being investigated [72].

**Figure 2:**
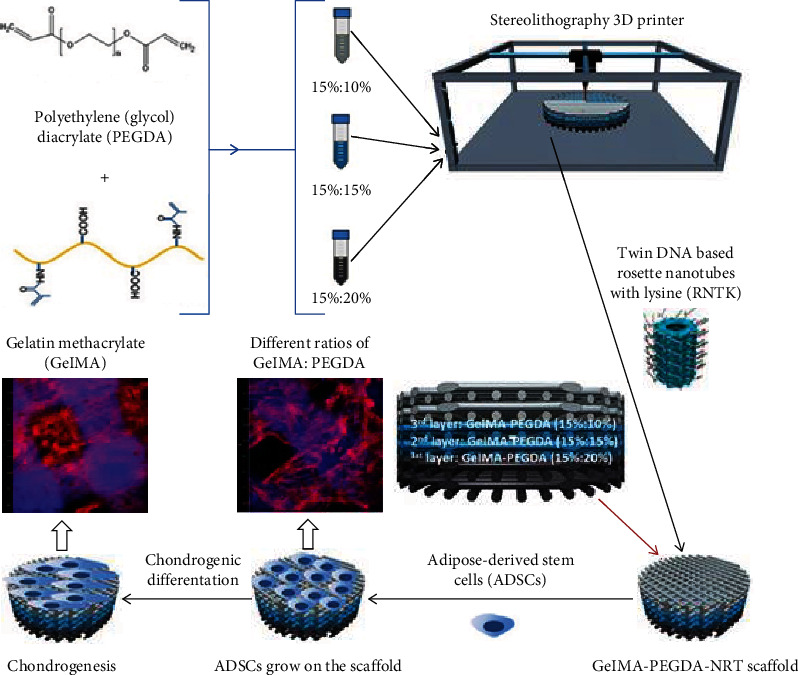
3D-printing hydrogel coated by DNA-based nanotubes [74].

Recently, multidisciplinary effort has been put into the fabrication of scaffolds that can specifically and molecularly interact with the cartilage microenvironment. Biological signals such as growth factors and signals are often incorporated into the hydrogels to enhance chondrogenesis in large cartilage defects [73]. For example, Zhou et al. [74] synthesized gelatin methacrylate (GelMA) and poly(ethylene glycol) diacrylate (PEGDA) and added DNA-based analogs to improve chondrogenesis of adipose-derived stem cells (ADSC). As shown in Figure 2, they printed a three-gradient scaffold, cultured ADSCs on it, and observed chondrogenic differentiation of ADSCs as well as ECM formation [74]. As another example, Kisiday et al. [75] devised a self-assemble peptide hydrogel scaffold to encapsulate chondrocytes. The molecularly engineered peptide used in their study provides tailored degradation rate and induced accumulation of cartilage-like ECM. Moreover, the manufactured hydrogel peptide provided the potential for tethering of growth factors and targeted delivery of growth factors to the chondrocytes [75].

The mechanical properties of hydrogels are considered their main limitation for load-bearing tissues [76]. Different methods have been used to address the poor mechanical properties of hydrogels. For example, Mohabatpour et al. [77] produced electrospun fiber polylactic acid (PLA) and grafted hyaluronic acid and alginate to enhance weight ratio nanofibers in composite to promote mechanical properties [77]. Similarly, Fenbo et al. [78] synthesized strontium alginate/chondroitin sulfate (Alg/CS-Sr) hydrogel with tunable stiffness by changing the concentration of strontium chloride. They inserted the hydrogel in the rabbit cartilage defect model and confirmed cartilage regeneration [78].

## 4. Hydrogel for Cartilage Tissue Regeneration

Hydrogels are good candidates for the construction of three-dimensional structures due to their tunable biomechanical properties and biocompatibility. Hydrogel scaffolds can regulate different materials to promote cartilage repair [11]. The hydrogel can be modified with cell adhesion ligands and its internal aqueous environment protects the cell and allows nutrient transportation [13].

Hydrogels can be used as filling agents, a delivery vehicle for bioactive molecules, and a three-dimensional structure for organizing cells [13, 88]. Different synthetic and natural polymers have been used for the fabrication of scaffold hydrogel for bone tissue engineering [89]. Although cartilage tissue is not successful in self-repair, recent hydrogel fabrication techniques are promising. For example, PLGA-gelatin/chondroitin/hyaluronate hydrogel scaffold was seeded with MSC, enhancing the MSC proliferation and GAG synthesis [90, 91]. Then, autologous differentiated MSC/PLGA-GCH was implanted in rabbit contralateral cartilage defect and it demonstrated better chondrocyte morphology and integration of continuous subchondral bone [13].

In order to generate MSC-based functional hyaline cartilage, MSCs should be directed to chondrogenic lineage and initiate the formation of cartilage matrix including collagen type II and glycosaminoglycan (GAGs) [90]. Although chondrogenic chemicals or growth factors have been used for chondrogenic differentiation of MSCs, protein denaturation, probability of carrying a pathogen, and undesired side effects limit their application [67, 92].

There have been several types of research to expand and improve the hydrogel that induces chondrogenesis; however, researchers are looking for physical cues that can promote cartilage regeneration after hydrogel is added [64]. The electromagnetic hydrogels are the new generation of hydrogel for cartilage tissue generation that is shown to be successful in in vitro and in vivo studies. These hydrogels have not still reached clinical practice and their long-term results have not been investigated yet. This review is focused on the hydrogels that induce cartilage regeneration through electromagnetic properties.

## 5. Magnetic Hydrogels for Cartilage Tissue Regeneration

The electromagnetic field (EMF) and pulsed electromagnetic field (PEMF) are FDA-approved techniques that are previously shown to promote osteogenic differentiation of MSC [17]. Human mesenchymal stem cells are widely accepted seeding cells and control of them by physical cues is of great interest in regenerative medicine [93]. Jiang et al. studied the uptake of magnetic nanoparticles by MSCs under the magnetic field. Iron-oxide nanoparticles-loaded bovine serum albumin (BSA) internalized by MSCs increased osteogenic differentiation and expression of collagen type I and osteocalcin significantly [94]. Several groups have reported that electromagnetic field promotes cartilage formation in vitro and in vivo. For example, adipose-derived stem cells were cultured in a hyaluronan microenvironment and treated with a pulsed electromagnetic field (PEMF) [95]. It was shown that under chondrogenic induction, PEMF stimulation promoted the expression of main chondrogenic genes such as SOX-9, collagen II, and aggrecan [92]. Similarly, Hou et al. [11] incorporated SPIONs into hyaluronic acid-graft-amphiphilic gelatin hydrogel and injected it into the rabbit's knee to study chondrogenic commitment. "They observed magnetic field upregulated Col II and SOX9 gene expression"" and magnetic derived" should be omitted. [11].

Therapeutic applications of the magnetic field keep expanding because magnetic nanoparticles can apply remote magnetic-induced physical stimulation which enables targeting of the specific site as well [11]. Mesenchymal stem cells labeled with magnetic nanoparticles were shown to induce higher differentiation [96]. In one study, human bone marrow-derived MSC was under static magnetic field and magnetic-derived shear stress via magnetic nanoparticles. It was demonstrated that biophysical stimulation resulted in higher chondrogenic differentiation efficiency [11].

It is shown that an early essential step for initiating the chondrogenic differentiation of the stem cell is its condensation [97]. For example, a decrease in intercellular spaces in the area of the cartilage and bone formation precedes cartilage differentiation during limb skeletogenesis [98]. In one study, magnetic labeling of the stem cell with the maghemite citrate-coated iron oxide resulted in cell condensation into aggregates. The magnetically cellularized scaffolds were exposed to transduction and shear stress stimuli produced high collagen type II [97]. The combination of magnetic cell seeding with dynamic differentiation induces chondrogenic differentiation as well as the creation of a millimeter-sized cartilage cellular construct [88]. Similarly, in another study [14, 99], Maghemite nanoparticles were used for labeling MSCs, and magnetic cells were suspended in chondrogenesis culture medium. Gene expression and histological studies proved that major cartilage matrix proteins (collagen II and aggrecan) were elevated [67, 88].

Magnetic nanoparticles can induce chondrogenic differentiation under the magnetic field when they bind to the cell surface. For example, Pulse electromagnetic fields (PEMFs) was applied to bone marrow mesenchymal stem cell (BMSC) cultured on the electromagnetic hydrogel. The hydrogel composed of gelatin, beta-cyclodextrin (beta-CD), and magnetic iron oxide (Fe_3_O_4_) induced the expression of late chondrogenic differentiation markers including *COL2* and *aggrecan* [9]. In addition, the hydrogel was implanted in rabbit knee cartilage defect revealing regenerative tissue that has completely filled the gap, and the histology staining was similar to that of natural cartilage.

Superparamagnetic iron oxide nanoparticles (SPIONs) can guide cells and serve as physical stimulation. In one study, SPIONs were encapsulated in a hydrophobic shell of hyaluronic acid-graft-amphiphilic gelatin (HA-AGMCs) microsphere. Hyaluronic acid is one of the major components of the chondrocyte extracellular matrix (ECM) which provides a backbone for aggrecan aggregation [11]. Furthermore, it interacts with the CD44 receptor to regulate signal transduction, cell migration, and differentiation [11]. Applying a magnetic field to the SPIONs incorporated into the (HA-AGMC) microsphere led to the expression of CoI II and SOX9 which are cartilage tissue-specific genes. In addition, this novel platform initiated chondrogenesis and sGAG synthesis [11].

In another study, magnetic nanoparticle-vesicle (MNVP) was assembled by cross-linking of phospholipid vesicles and magnetite nanoparticles. Chondrocytes and nanoparticle-vesicle assemblies were coimmobilized within a calcium alginate hydrogel. This smart biomaterial responds to the alternating magnetic field by translating noninvasive magnetic signals into cellular responses [13]. Their research proved that the chondrocyte in the gel responded to the magnetic release of ascorbic acid-2-phosphate (AAP) which was applied as an additive by producing a high level of collagen [13]. Similarly, in another study, dextran-coated magnetic nanoparticles were integrated into the distinct layer of agarose construct to create trilayered ferrogel [100]. An external magnetic field of the 0.5 T was applied to the bovine chondrocytes seeded in ferrogels demonstrating that sGAG content increased over time [100].

The other advantage provided by the incorporation of magnetic nanoparticles into the hydrogel is they can serve as a contrast agent for imaging [101]. Magnetic resonance imaging (MRI) is a noninvasive technique that enables longitudinal imaging at successive time points and can be used to visualize the molecular changes and remodeling of the repairing tissue [101, 102]. Yang et al. [101] incorporated SPIONs-Kartogenin into cellulose nanocrystal/dextran hydrogel. The SPION-labeled hydrogel not only induced chondrogenesis both in vitro and in vivo but also demonstrated magnetic resonance contrast enhancement [101]. Similarly, Chen et al. [103] fabricated USPIO-labeled cellulose nanocrystal (CNC)/silk fibroin (SF) composite hydrogel which enhanced chondrogenic gene upregulation in vitro as well as provided a mean for measuring hydrogel degradation through MRI imaging [103].

### 5.1. Methods for Synthesizing and Characterizing Magnetic Hydrogels

The cartilage microenvironment can be triggered by magnetic stimuli through being exposed to the external magnetic field or magnetic scaffolding [104]. Approaches used in order to synthesize the magnetic scaffolds include both conventional and modern techniques [105]. The conventional method of fabrication of magnetic hydrogel includes mixing nanoparticles with hydrogel, precipitation, blending, and grafting method [105], while the recent research has focused on additive manufacturing. Farzaneh et al. [104] mixed cobalt ferrite nanoparticles (CFNs) with hydrogel precursors to produce magnetic hydrogel. The magnetic properties of a synthesized hydrogel such as hysteresis curve were studied by vibrating sample magnetometer (VSM) instrument [104]. Furthermore, Huang et al. [106] added the iron oxide magnetic nanoparticles as embedment to the gelatin and *β*-cyclodextrin and inserted them into the rabbit cartilage defect. MSCs seeded in hydrogel exposed to the PEMF promoted the differentiation of the stem cells by promoting COL2 and aggrecan [106]. Similarly, Zhang et al. [107] first coprecipitated the iron oxide nanoparticles with polyvinyl alcohol and then synthesized it with the combination of collagen II, polyethylene glycol, and hyaluronic acid to fabricate magnetic hybrid gel which mimics the natural cartilage ECM [107].

**Figure 3:**
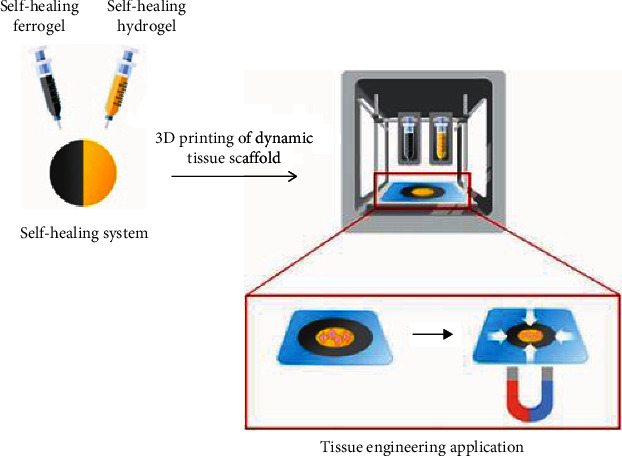
3D printing of scaffold including self-healing hydrogel and ferrogel [109].

The additive manufacturing technique is promising for the fabrication of magnetic hydrogels due to their potential to be customized and be reproduced [108]. De Santis et al. [108] synthesized polycaprolactone-polyethylene glycol-based magnetic scaffold by stereolithography approaches for articular cartilage tissue regeneration [108]. Also, Choi et al. [109] fabricated a scaffold composed of self-healing hydrogel combined with ferrogel via extrusion 3D printing technique as demonstrated in Figure 3. The ferrogel demonstrated superparamagnetic properties due to containing SPIONs. They conducted in vitro study by exposing the cells encapsulated in hydrogel to the external magnetic field. They showed that the magnetic stimulation upregulates the SOX-9 and COL-2 which indicates the potential of fabricated magnetic hydrogel for cartilage tissue regeneration [109].

### 5.2. Underlying Biological Mechanism of Enhanced Chondrogenesis Dependent on Magnetic Stimuli

Magnetic hydrogels can be used to influence cellular mechanotransduction and translational and paracrine responses of the cells to encourage cartilage repair through different cellular and molecular mechanisms [19]. The mechanisms by which magnetic signals promote cartilage include enhancing cell-cell interaction, MSC adherence to the defect area, and activating mechanosensitive channels [110]. For instance, Kamei et al. [111] proved that cell adhesion molecules such as integrin *α*2 (ITG *α*2), integrin *α*6 (ITG *α*6), integrin *β*3-binding protein (ITG *β*3BP), intercellular adhesion molecule–2 (ICAM-2), and platelet/endothelial cell adhesion molecule–1 (PECAM-1) have been upregulated in magnetically labeled MSCs. Increased adhesion rate of MSCs can make them engraft to the defect area biologically [111].

Also, the electromagnetic field can induce cartilage tissue regeneration by stimulating chondrocyte maturation [112–115]. For instance, Yi et al. reported that magnetic field upregulates the chondrogenic genes in stem cells and also enhances the synthesis of proteoglycans. They suggested that this might be due to increased expression of transforming growth factor beta in presence of magnetic field. Yan et al. [117] showed that increased cartilage regeneration under EMF is associated with enhanced (TGF-*β*) and it results from the activation of the Wnt1/LRP6/*β*-catenin signaling pathway [117]. Moreover, magnetic signals have been shown to contribute to chondrogenesis by activating calcium-permeable transient receptor potential (TRP) channels [19]. Parate et al. [19] have demonstrated that paracrine activity of MSC secretome provides the potential for chondrogenic differentiation of MSCs when they are exposed to the magnetic signal [19].  Table 2 summarizes the advantages and disadvantages of different cartilage repair strategies.

## 6. Future Directions and Limitations

Magnetic hydrogels have been shown to be promising for the treatment of cartilage lesions due to their influence on the stem cell fate as well as chondrocyte behavior [14]. Magnetic hydrogels can provide other advantages such as being used for delivery and timely release of the growth factors. In addition, magnetic strength in tissue formation controls the processes influencing interface regeneration and the homeostasis [118]. Recent research proved the application of electromagnetic field promotes progress of the fascinating line in the field of regenerative medicine for cartilage tissue regeneration. Moreover, magnetic nanoparticles can manipulate the microstructure by noncontact magnetic forces or inducing mechanical stresses at the microscopic level through applied magnetic field which may result in cell proliferation and differentiation [16]. However, these hydrogels still struggle with cell differentiation [11] and they need improvements to generate a more biomimetic and functional cartilage substitute for future preclinical applications [16].

## Figures and Tables

**Table 1:** Most common polymers for cartilage hydrogel scaffolds and their biological activities.

Polymer	Properties	References
Collagen	It has chondro-inductive properties which lead to a suitable 3-D microenvironment for enhancing MSC chondrogenesis. Collagen can also provide immunomodulatory properties by reducing certain immunogenic effects.	[28–30]
Chondroitin sulfate	It provides chondro-protective and anti-inflammatory properties to enhance cartilage tissue regeneration. Furthermore, it increased the production of collagen type II.	[23]
Hyaluronic acid (HA)	It improves early-stage chondrogenesis and was proved to repair osteochondral defects in vivo studies. HA interacts with receptors such as CD44 to adjust signal transduction and stem cell differentiation.	[11, 31, 32]
Alginate	It enhances the proliferation of chondrocytes and maintains the chondrocyte phenotype. Its fast and simple gelation makes it suitable for injection.	[33, 34]
Chitosan	The similarity of its structure with GAG leads to chondrocyte proliferation and chondrogenesis. It also improves chondrocyte homeostasis.	[35, 36]

**Table 2:** Advantages and disadvantages of different cartilage repair strategies.

Technique	Advantages	Disadvantages	References
Microfracture technique	It is minimally invasive and there is no need for a tissue graft.	It does not restore normal hyaline cartilage and leads to losing undamaged cartilage. This technique is just applied for the lesion size less than 2.5 cm^2^.	[53, 55, 79]
Osteochondral transfer	Autograft provides a fresh viable cartilage tissue from the patient. Allograft is a useful technique for different defect sizes and locations. Also, it decreases the operation time compared with autograft.	Autograft may lead to donor-site morbidity. Also, some patients do not have proper donor tissue. Moreover, the autograft cannot be normally used for repairing large defects. Allograft may lead to graft host reaction.	[80–84]
ACI	Can repair large cartilage defects with minimum donor-site morbidity	It leads to periosteal hypertrophy and graft delamination	[55, 85]
Scaffold	High biocompatibility, incorporating growth factors and tunable properties		[86, 87]

## Data Availability

SPSS data of the participant can be requested from the authors. Please write to the corresponding author if you are interested in such data.
